# Molecular Characterization and Genotype-Phenotype Correlation of G6PD Mutations in Five Ethnicities of Northern Vietnam

**DOI:** 10.1155/2022/2653089

**Published:** 2022-07-05

**Authors:** Thi Thao Ngo, Thinh Huy Tran, Thanh Dat Ta, Thi Phuong Le, Phuoc Dung Nguyen, Mai Anh Tran, The-Hung Bui, Thanh Van Ta, Van Khanh Tran

**Affiliations:** ^1^Center for Gene and Protein Research, Hanoi Medical University, Hanoi 10000, Vietnam; ^2^Biochemistry Department, Hanoi Medical University, Hanoi 10000, Vietnam; ^3^Hanoi Medical University Hospital, Hanoi Medical University, Hanoi 10000, Vietnam; ^4^Center for Molecular Medicine and Surgery, Clinical Genetics Unit, Karolinska Institute, Karolinska University Hospital, Stockholm 14186, Sweden

## Abstract

Glucose-6-phosphate dehydrogenase (G6PD) deficiency is the most common enzyme disorder and is caused by G6PD gene mutations. To date, more than 400 variants in the G6PD gene have been discovered, and about 160 identified variants are associated with a significant decrease in the G6PD enzyme activity. However, the molecular characterization and epidemiological study of G6PD deficiency are still limited in Vietnam. Therefore, we conducted this study to determine the G6PD variants among the Vietnamese populations and evaluate their correlation to G6PD enzyme activity. A total of 339 patients (302 males and 37 females) were enrolled in this study. The G6PD variants were identified by Sanger sequencing. Our results indicate that males are more severely deficient in G6PD than females. This enzyme activity in males (1.27 ± 1.06 IU/g·Hb) is significantly lower than in females (2.98 ± 1.57 IU/g·Hb) (*p* < 0.0001). The enzyme activity of the heterozygous-homozygous females and heterozygous females-hemizygous males was found to be significantly different (*p* < 0.05), which is interpreted due to random X-inactivation. For G6PD molecular characteristics, *Viangchan* (c.871G>A), *Canton* (c.1376G>T) and *Kaiping* (c.1388G>A) variants were the most dominant, accounting for 24.48%, 17.70%, and 22.42%, respectively, whereas the highest frequency of complex variants was observed in *Viangchan/Silent* with 20.35%. In terms of G6PD activity, the *Union* variant presented the lowest mean value (1.03 IU/g·Hb) compared to the other variants (*p* < 0.05). Computational analysis using Polyphen-2 tool investigated that all variants were relative to G6PD deficiency and separated the levels as benign and damaged. The result will establish effective methods to screen G6PD variants in Vietnam.

## 1. Introduction

Glucose-6-phosphate dehydrogenase (G6PD) is a key cytosolic enzyme in the pentose phosphate pathway (PPP) to produce NADPH which plays an important role in protecting the red blood cells from oxidative stress by reducing glutathione dimers oxidized and sulfhydryl groups [1]. The lack of the G6PD enzyme can cause hemolytic and related disorders such as clinical acute hemolysis, neonatal jaundice, and congenital hemolytic anemia [2,3]. G6PD deficiency, also known as favism, is the most prevalent enzyme disorder and is found worldwide [4–6]. An estimated 400 million people were influenced globally by G6PD, and an average of 4100 people died every year from 1990 to 2013 [7]. Based on the residual enzyme activity and clinical manifestations, G6PD deficiency is categorized into five groups by the WHO. Class I (less than 1% of normal activity) has been considered the most serious among classes and is specifically associated with chronic nonspherocytic hemolytic anemia (CNSHA). Class II (1 to 10% of normal) is highly associated with acute hemolytic anemia, while Class III (10 to 60% of normal) is normally associated with occasional acute hemolytic anemia, and Class IV (60 to 150% of normal) and Class V (>150% of normal) are mostly asymptomatic [8].

G6PD is encoded by the G6PD gene which is located in the telomeric region of the X chromosome (Xq28). Thus, G6PD deficiency has been inherited as the X-linked incomplete dominant. While males are always hemizygous because of having only one X chromosome, females with this disorder may be heterozygous or homozygous and have less severe clinical manifestations [9]. The G6PD gene is 18.5 kb in size with 13 exons and codes for 515 amino acids of the G6PD enzyme. To date, more than 400 variants in the G6PD gene have been discovered, and about 160 identified variants show a significant decrease of the enzyme in erythrocytes [10,11]. The vast majority of G6PD variants are single-base substitutions and are distributed as follows: 85.4% are missense, 8% are multiple mutations, 5.3% are deletions, and 1% are mutations within introns [12]. Moreover, many variants present genetic characteristics within specific populations, geographic regions, and ethnic groups [13]. For example, the *G6PD Mediterranean* (c.563C>T) variant is widely distributed in Southern Europe, the G6PD A-variant is predominant in African origins, and the G6PD *Mahidol* (c.487G>A) and *Viangchan* (c.871G>A) variants are mostly associated with Asians, especially in Myanmar and Cambodian populations [14–16]. Furthermore, the correlation between these variants and the deficiency of the G6PD enzyme is being investigated. The G6PD variants such as *Canton* (c.1376G>T)*, Kaiping* (c.1388G>A), and *Gaohe* (c.95A>G) have been identified to reduce enzyme activity by up to 90% in the Chinese population. Different variants can cause varying enzyme activities [17,18].

In Vietnam, G6PD deficiency is also a prevalent genetic disorder with an incidence rate of about 8.9% and a diverse distribution according to ethnic groups and regions [19,20]. Several G6PD variants are characterized by being detected in the Vietnamese population, such as *Vietnam1* (c.7G>A), *Vietnam2* (c.197T>G), and *BaoLoc* (c.352T>C) [21,22]. However, the correlation between specific G6PD variants and these activity genotypes has not been reported. Also, the molecular epidemiology of G6PD deficiency is still limited in Vietnam. To provide more information to diagnose this disorder, we performed this study to carry out the prevalence of G6PD deficiency and G6PD variants in the Vietnamese population by direct sequencing. These data will contribute to prenatal genetic counseling to reduce morbidity, reduce consequences for the patient's family and society, and improve the quality of health care in the community.

## 2. Materials and Methods

### 2.1. Sample Collection

To screen G6PD variants, 339 pediatric patients were selected from 25 provinces of Northern Vietnam and confirmed G6PD deficiency with an enzyme activity less than 6 IU/g·Hb by Vietnam National Children's Hospital from 2017 to 2020. The patients belonged to five different ethnic groups: Kinh, Mong, Muong, Thai, and Tay. The ages were arranged between 1-month-old and 24-month-olds. The participants consented to enroll before the study´s commencement. Whole blood samples were obtained in K2-EDTA tubes with a concentration of 1.5 mg/mL, then the G6PD enzyme activity was measured by an automated biochemistry analyzer AU5800/AU680 (Beckman Coulter, USA) at the Department of Biochemistry, Vietnam National Children's Hospital.

### 2.2. Molecular Characteristic Analysis of G6PD Variants

Genomic DNA was extracted from peripheral blood samples by following the Wizard Genomic DNA purification kit instruction (Promega, USA). The primers for amplifying the G6PD gene were designed according to Nguyen Thi Hue et al. (2009) with minor modifications [21]. For PCR, the mixture contains GoTaq Hot Start Master Mix (2X), primer set (1 *μ*M), DNA template (50 ng/*μ*L), and sterile water. The PCR conditions were performed by initial denaturation at 95^o^C for 2 min, followed by 35 cycles of denaturation at 95°C for 30 s, annealing at 60°C for 30 s, and extension at 72°C for 30 s, followed by a final extension at 72°C for 5 min then holding at 4^o^C. After purification, PCR amplicons were sequenced by an ABI 3500xl Genetic Analyzer (Applied Biosystems, France). To identify G6PD variants, the sequencing results were analyzed by CLC Main Workbench software and assembled with the G6PD sequence on GenBank (NG_009015).

### 2.3. Damaging In Silico Analysis

The estimated damage score was evaluated by using the PolyPhen-2 web server (genetics.bwh.harvard.edu/pph2/index.shtml) [23]. For PolyPhen-2, the predicted function of a variant is classified as benign, possibly damaging, or probably damaging, with the scale score arranged from 0 to 0.5, 0.5–0.9, and 0.9–1, respectively. The G6PD query protein sequence from UniProtKB (P11413) was mapped as a reference.

### 2.4. Statistical Analysis

Statistical analysis was evaluated by GraphPad Prism ver.9 software. Comparison among groups was conducted using one-way ANOVA. Variables such as age, detection rate, and genotype were described by descriptive statistics. A chi-square test was applied for the comparison of frequencies of G6PD deficiency between both genders. A *p*value < 0.05 was considered statistically significant.

## 3. Results

### 3.1. Patient Clinical Characteristics

**Figure 1:**
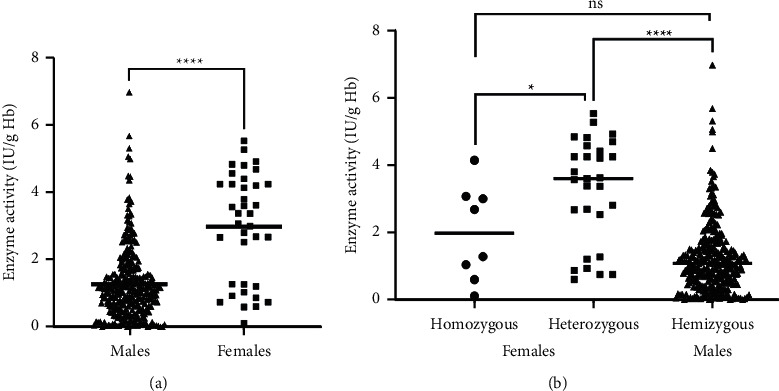
Comparative distribution of G6PD activity by genders and genotypes. Each dot represents the G6PD enzyme activity of each subject. (a) G6PD activities between males and females. (b) G6PD activities among genotypes in both males and females.

A total of 339 patients were enrolled in this study, which contained 302 males (89.09%) and 37 females (10.91%) from five different ethnicities: Kinh, Muong, Nung, Thai, and Tay. The participant characteristics are listed in Table 1. The infants' age was distributed from less than 1-month old (79.06%) to over 24-months old (0.88%). There was a significant difference in age between males and females (*p*=0.0089) (Table 1). The G6PD enzyme activity was measured as 1.46 ± 1.24 IU/g·Hb on average. A significant difference was valued between both genders with 1.27 ± 1.06 IU/g·Hb in males and 2.98 ± 1.57 IU/g·Hb in females (*p* < 0.0001) (Figure 1(a)). In normal conditions, the G6PD reference range at 37^o^C is 6–20.5 IU/g·Hb. The level of G6PD deficiency was categorized as high level (<0.6 IU/g·Hb) with 79 patients (23.30%), medium level (0.6–3.6 IU/g·Hb) with 233 patients (68.74%), and low level (3.6–9 IU/g·Hb) presented in 27 patients (7.96%). There was a significant difference in these levels between genders (*p* < 0.0001). Among 339 cases, 332 cases were found to carry G6PD genetic variants in both males and females including homozygous (2.36%), hemizygous (85.84%), and heterozygous (8.55%) (*p* < 0.0001). No G6PD variants were recorded in 11 cases (3.25% in males) (Table 1).

### 3.2. Prevalence of the G6PD Enzyme Activity in Five Ethnic Groups

**Figure 2:**
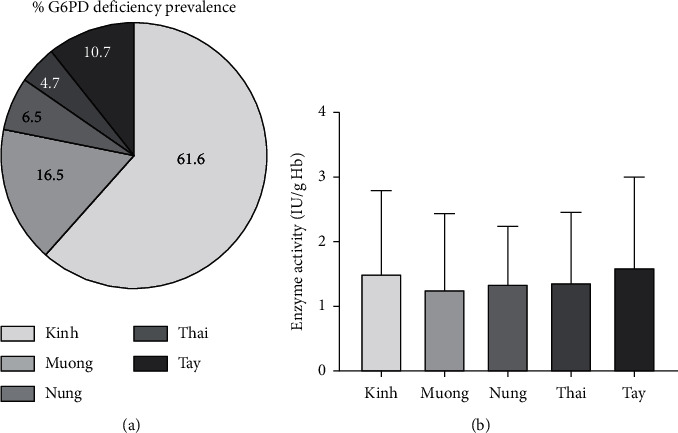
The prevalence and G6PD enzyme activity among Vietnamese ethnics. (a) The prevalence of G6PD deficiency among five different ethnicities. (b) Distribution of G6PD activities according to ethnic groups.

In our study, the five selected ethnicities were distributed in Northern Vietnam. The majority of samples were arranged in Kinh, followed by Muong, Tay, Nung, and Thai with different prevalences of 61.6%, 16.5%, 10.7%, 6.5%, and 4.7%, respectively (Figure 2(a)). Also, the Tay population presented the highest enzyme activity (1.61 ± 1.37 IU/g·Hb), and the lowest enzyme activity was observed in the Muong population (1.26 ± 1.16 IU/g·Hb) (Figure 2(b)). However, no significant difference was recorded between the enzyme activity and ethnic groups (*p*=0.6487).

### 3.3. Identification and Function Prediction of G6PD Variants

**Figure 3:**
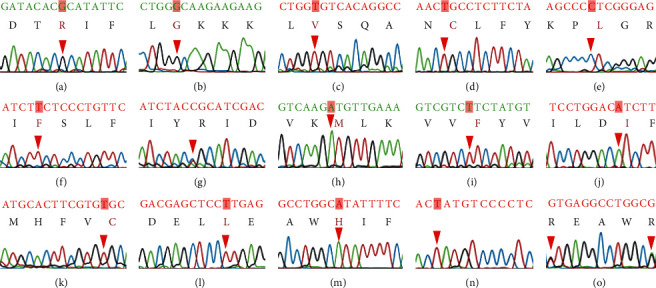
The electropherogram of G6PD variants. (a) Gaohe (c) 95A > G), (b) Orissa (c.131 C > G), (c) Quing Yan (c.392 G > T), (d) Valladolid (c.406 C > T), (e) NanKang (c.517 T > G), (f) Mediterranean (c.563 C > T), (g) Coimbra Shunde (c.592 C > T), (h) Viangchan (c.871 G > A), (i) Chinese-5 (c.1024 C > T), (j) Taiwan-2 (c.1330 G > A), (k) Union (c.1360 C > T), (l) Canton (c.1376 G > T), (m) Kaiping (c.1388 G > A), (n) Silent (c.1311 C > T), and (o) Canton/Kaiping (c.1376 G > T/c.1388 G > A).

With 339 participants, 14 G6PD variants were detected by using the Sanger sequencing method and categorized into two types: missense and silent (Table 2) (Figure 3). Of these, the *Viangchan* (c.871G>A), *Canton* (c.1376G>T), and *Kaiping* (c.1388 G > A) variants were the most dominant across the five ethnic groups, accounting for 24.48%, 17.70%, and 22.42%, respectively (Figures 3(h), 3(m), 3(n)). A silent variant (c.1311C>T) in exon 11 was also found with a high frequency (25.66%) (Figure 3(o)). Moreover, the *NanKang* (c.517 T > G), *Mediterranean* (c.563C>T), *Coimbra Shunde* (c.592C>T), and *Taiwan-2* (c.1330G>A) variants were relatively rare and were only detected in one sample each (0.29%) (Figures 3(e), 3(f), 3(g), 3(j)). In addition, the coexistent variants were found in our samples with variable frequencies, mostly together with *Silent* variants (c.1311C>T), presented in *Valladolid/Silent* variant (0.59%), *Viangchan/Silent* variant (20.29%), *Union/Silent* variant (2.06%), *Canton/Silent* variant (0.59%), and *Kaiping/Silent* variant (0.88%) (Table 2). A unique variant between *Canton* and *Kaiping* was found in one tested individual (0.29%) (Figure 3(o)).

PolyPhen-2 is a useful automatic tool for the prediction of the possible impact of an amino acid substitution on the structure and function of a human protein [23]. In this study, computational analysis was performed to estimate the risk of disease among G6PD variants. A total of 14 variants, four of them were identified to have the maximum damaging score (DS = 1) including *Valladolid*, *NanKang*, *Coimbra Shunde*, and *Union*, and the benign score was observed in *Mediterranean* (0.371), *Chinese-5* (0.205), and *Taiwan-2* (0.127) (Table 2). A high-risk score was also accessed in the remaining variants, arranging from 0.860 to 0.998. The *Silent* variant (c.1311C>T) did not give any score by Polyphen-2 because it was a silent variant.

### 3.4. Correlation between the Genotype and the G6PD Activity Phenotype

**Figure 4:**
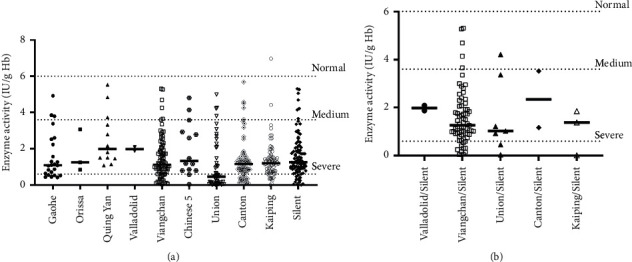
Distribution of G6PD activities according to mutation types. Only variant groups in which there were ≥ 2 representatives are shown. (a) G6PD activities of single identified variants. (b) G6PD activities of compound variants.

According to the WHO instruction, the 13 identified G6PD variants in our study were predominantly identified in Class II and III, except for the silent variant. Among these variants, *Gaohe*, *Orissa*, *Quing Yan*, *Chinese-5,* and *Taiwan-2* were categorized as Class III, while *Valladolid*, *NanKang*, *Mediterranean*, *Coimbra Shunde*, *Viangchan*, *Union*, *Canton*, and *Kaiping* were categorized as Class II (Table 3). The G6PD variant genotype was mainly found in hemizygous (294/332) males (1.26 ± 1.04 IU/g·Hb), while homozygous (8/332) and heterozygous genotypes (29/332) were commonly observed in females with the enzyme activity arranged 1.99 ± 1.33 IU/g·Hb and 3.26 ± 1.52 IU/g·Hb, respectively. The lower activity was significantly observed in hemizygous males than in heterozygous females (*p* < 0.0001), whereas a statistical difference was evaluated when a comparison of activity between homozygous females and heterozygous females was made (*p*=0.04) (Figure 1(b)). In terms of G6PD activity, the *Union* variant presented the lowest mean value (1.03 IU/g·Hb), followed by *Canton* (1.4 IU/g·Hb) and *Kaiping* variant (1.35 IU/g·Hb) (Figure 4(a)). Variant groups in which there were ≥2 representatives were shown in Figure 4. There was a significant difference between these variants and the enzyme activity (*p*=0.0088). In addition, the activity of the cooccurred variants was also presented, but no significant difference was recorded (*p*=0.8139) (Figure 4(b)). Among the coexistent variants, we found that only the *Viangchan/Silent* variants presented a correlation to the G6PD enzyme activity (*r*=0.3186, *p*=0.0033), while the other variants did not show the relationship.

## 4. Discussion

G6PD deficiency is a common enzyme abnormality that affects approximately 5% of the world´s population and causes some diseases related to erythrocytes [24]. Some mutations on the G6PD gene are being investigated to be associated with this deficiency of the G6PD enzyme activity. Therefore, identifying G6PD variants plays an important role in screening and estimating the risk variants in communities.

The distribution of G6PD deficiency is variable across ethnic groups and geographical regions [13]. In our study, the 339 blood samples were selected from five ethnicities in Northern Vietnam including Kinh, Muong, Nung, Thai, and Tay. The results showed that while the Kinh ethnic group had the highest prevalence of G6PD deficiency (∼60%), the Thai ethnic group carried the lowest G6PD distribution compared to the others (Figure 2(a)). The main distribution of G6PD deficiency in the Kinh ethnic group can be explained by the predominance of this ethnic group in Vietnam, accounting for approximately 86% of the population [25]. However, no statistically significant difference was observed between the G6PD incidence and ethnic groups (*p* > 0.05). Similar results were reported in the Kinh and S'Tieng ethnic groups, according to Nguyen Thi Hue et al. (2009). Although the frequency of G6PD deficiency in Southern Vietnam was rather high, accounting for 11.3%, this proportion in the Kinh and S'Tieng populations was only 8.7% and 14%, respectively (*p*=0.07) [21]. Likewise, compared to Myanmar ethnicities, the Kachin people have a higher level of G6PD prevalence (29.6%) compared to other local groups such as Mon (12%), Burmese (10%), Karen (12.9%), and Burman [26–28]. Also, the variable of G6PD distribution was observed among the Lue ethnicities in Thailand. Although the rate of G6PD in the Lue ethnic group was 13.51%, the different local languages show the variation in Ta-Kadai (9.69%), Sino-Tibetan (4.51%), Austroasiatic (7.58%), and Hmong-Mien groups (1.77%) [29]. An extreme distribution of G6PD deficiency in the Great Mekong Subregion (GMS) countries can be understood because these countries were seriously affected by the malaria pandemic [30]. It could be a possible evolutionary factor to increase the prevalence of G6PD deficiency in the population.

Furthermore, our results indicated that males were more severely deficient in G6PD than females. This enzyme activity in males (1.27 ± 1.06 IU/g·Hb) was significantly lower than in females (2.98 ± 1.57 IU/g·Hb) (*p* < 0.0001) (Table 1; Figure 1(a)). Because the G6PD gene is located on the X chromosome, its expression can be different between both genders. Males have only one X chromosome and will be hemizygous with G6PD mutations [31]. Therefore, G6PD deficiency can express fully in this phenotype compared to that in females, which can be caused by X-inactivation [32, 33]. Females with G6PD heterozygous genotypes present a wide range decrease of G6PD activity, a range from 20–80% with the normal [34].

In this study, we identified 13 G6PD variants by Sanger sequencing. The majority of variants are chiefly discovered in China, India, and they have been established as Asia variants; the other is found in European origin countries and Mediterranean areas such as *Valladolid* and *Mediterranean* [12,13]. The finding supports the notion that the genetic drift event occurred in the Asian population in the prehistoric period. For example, the migration of Chinese to Vietnam has been recorded for a long time and gradually Vietnamized to be Hoa ethnics [35]. Ethnic migration is investigated to play a crucial role in regular genetic trait distribution and the characteristics of populations according to the gene flow process [36–38]. In Yuzhong Zhen's study, the heatmap for distribution of the G6PD-deficient allele indicates that Canton, Kaiping, and Gaohe are highly related to the Chinese population, the G6PD Viangchan and Mahidol were mostly related to the Southern Asian population. The ethnic migration suggested that the Chinese variants occurred before the formation of these Chinese ethnic populations [39].

Of 13 G6PD variants, we found that *Viangchan* variants had the highest frequency among our ethnic groups with 24.48% (Table 1). It has been considered the most common mutation in Asia with diverse distribution between regions and ethnicities. In Southern Vietnam, this variant is highly detected in the Kinh and K'Ho ethnic groups with 44%, and 75% is observed in the Raglai and Pako ethnic groups [22, 40]. In several countries of GMS, the *Viangchan* variant is found in Laotians (100%), Cambodians (97.9%), and Thais (67.7%) [16, 41, 42]. The sharing of G6PD *Viangchan* among Southeast Asian populations reveals insight into old ancestral sources in these countries. In addition, the *Canton* and *Kaiping* variants are the most prevalent in South West China with 20% and 79.16%, respectively, found in 17.7% and 24.2% of our samples [43]. Likewise, in Thailand, *Canton* and *Kaiping* are observed in 15.4% and 14.4% of G6PD deficiency cases, respectively [5]. The G6PD *Union,* which was presented at 15.04% in this study, is determined at 100% in the Khomu population and 9.5% in Thailand [20, 44]. On the other hand, G6PD *Gaohe* is also an important Chinese variant with an incidence rate ranging from 8.8% to 14.2% in different studies and was identified at about 7.08% in this study [45–47]. The Chinese variants including *Orissa* (0.88%)*, Quing Yan* (3.54%)*, NanKang* (0.29%)*, Taiwan-2* (0.29%)*, Chinese-5* (0.29%), and *Coimbra Shunde* (0.29%), and European variants such as *Valladolid* (0.59%) and *Mediterranean* (0.29%) were rarely detected in Northern Vietnam but were observed in several studies with various frequencies [5, 18, 29, 48–51]. Moreover, *Silent* variants are the most common polymorphism of G6PD gene and have a high rate of distribution among populations [18, 21, 29].

To date, *in silico* analysis has been applied to estimate the pathogenic mutations for disease. In the current study, the Polyphen-2 tool reported *Mediterranean*, *Chinese-5*, and *Taiwan-2* variants as benign, whereas the other variants were damaged, almost similar to the G6PD classification of WHO (Tables 2, 3). These results suggest that all G6PD variants can be caused by G6PD deficiency. The application of bioinformatics tools in G6PD mutations has been investigated in different populations [52–54]. In Chinese ethnicity, the lowest enzyme activity is G6PD *Canton* variant, which was recorded in the *Union* variant of our data [18]. In addition, the *Mediterranean* variant, which is considered more severe by the WHO classification, was found to be benign through bioinformatic analysis in this study. The different results can be understood by the variable of gene expression within and between populations [55]. Although the *Silent* variant is a silent mutation in the intronic region, it has been investigated relatively to G6PD deficiency [56]. Some hypotheses are postulated to interpret the expression of enzyme activity of this mutation. By predicting the secondary structure of G6PD mRNA, the mutant *Silent* presents the stable structure at the start codon boundary, therefore causing a negative effect on mRNA translation [57]. Or this single mutation may be located in the enhance region, where nucleotide alteration can change the function and lead to reduced gene expression [58]. Thus, further studies should be performed to clarify the mechanism of the *Silent* variant. Among coexistent variants, the linkage disequilibrium between G6PD *Viangchan* and *Silent* was the most common, occurring in Thai, Vietnamese, and Chinese populations [42, 58, 59]. However, the correlation of these covariants to the G6PD enzyme activity is not fully understood.

## 5. Conclusions

In this study, we successfully identified 13 G6PD variants related to G6PD deficiency in Northern Vietnam by Sanger sequencing. G6PD *Viangchan, Canton, Kaiping,* and *Union* variants were the most prevalent across Vietnamese ethnic groups, accounting for 79.64% of samples. In addition, the six cooccurred variants were also observed at different frequencies. The correlation between these single variants and G6PD deficiency was investigated by a bioinformatic tool, further studies should be performed on coexistent mutations. The result will contribute to the diagnosis and screening of G6PD deficiency in Vietnam, reduce consequences for the patient's family and society, as well as improve the quality of health care in the community.

## Figures and Tables

**Table 1:** Participant characteristics in this study.

	Male	Female	*Total*	*p*
*Age*				
<1 month	244 (71.98)	24 (7.08)	268 (79.06)	0.0089^*∗*^
1–6months	50 (14.75)	9 (2.61)	59 (17.41)	
>6–12 months	5 (1.48)	1 (0.29)	6 (1.77)	
>12–24 months	1 (0.29)	2 (0.59)	3 (0.88)	
>24 months	2 (0.59)	1 (0.29)	3 (0.88)	
N	**302**	**37**	**339**	

*Enzyme activity* (IU/g·Hb)				
Average	1.27 ± 1.06	2.98 ± 1.57		<0.0001^*∗*^

*The G6PD deficiency level of participants*				
High (<0.6 U/g·Hb**)**	78 (23.01)	1 (0.29)	79 (23.30)	<0.0001^*∗*^
Medium (0.6–3.6 IU/g·Hb)	212 (62.54)	21 (6.20)	233 (68.74)	
Low (3.6–9 IU/g·Hb)	12 (3.54)	15 (4.42)	27 (7.96)	
N	**302**	**37**	**339**	

*Genotype*				
No mutation	11 (3.25)	0	11 (3.25)	<0.0001^*∗*^
Homozygous	0	8 (2.36)	8 (2.36)	
Hemizygous	291 (85.84)	0	291 (85.84)	
Heterozygous	0	29 (8.55)	29 (8.55)	
N	302	37	339	

**Table 2:** Distribution of G6PD variants in this study.

Variant name	Position	Amino acid substitution	Type of variant	Exon	Case	Frequently (%)	In silico analysis
*Gaohe*	c.95A>G	H32A	Missense	2	24	7.08	Damaging score: 0.998
*Orissa*	c.131C>G	A44G	Missense	3	3	0.88	Damaging score: 991
*Quing Yan*	c.392G>T	G131V	Missense	5	12	3.54	Damaging score: 0.826
*Valladolid*	c.406C>T	L142C	Missense	5	2	0.59	Damaging score: 1
*NanKang*	c.517T>G	F173L	Missense	5	1	0.29	Damaging score: 1
*Mediterranean*	c.563C>T	S188P	Missense	5	1	0.29	Benign score: 0.371
*Coimbra Shunde*	c.592C>T	R198C	Missense	6	1	0.29	Damaging score: 1
*Viangchan*	c.871G>A	V291M	Missense	9	83	24.48	Damaging score: 0.996
*Chinese-5*	c.1024C>T	L342F	Missense	9	14	4.13	Benign score: 0.205
*Taiwan-2*	c.1330 G > A	V444I	Missense	11	1	0.29	Benign score: 0.127
*Union*	c.1360C>T	R454C	Missense	11	51	15.04	Damaging score: 1
*Canton*	c.1376G>T	R459L	Missense	12	60	17.70	Damaging score: 0.910
*Kaiping*	c.1388G>A	R463H	Missense	12	76	22.42	Damaging score: 0.860
*Silent*	c.1311C>T	T437T	Silent	11	87	25.66	N/A
*Valladolid/Silent*	c.406C>T	A142C	Missense	5, 11	2	0.59	N/A
	c.1311C>T	T437T	/Silent				
*Viangchan/Silent*	c.871G>A	V291M	Missense	9, 12	69	20.35	N/A
	c.1311C>T	T437T	/Silent				
*Union/Silent*	c.1360C>T	R454C	Missense	11	7	2.06	N/A
	c.1311C>T	T437T	/Silent				
*Canton/Silent*	c.1376G>T	R459L	Missense	11, 12	2	0.59	N/A
	c.1311C>T	T437T	/Silent				
*Kaiping/Silent*	c.1388G>T	R463H	Missense	11, 12	3	0.88	N/A
	c.1311C>T	T437T	/Silent				
*Canton/Kaiping*	c.1376G>T c.1388G>A	R459L, R463H	Missense	12	1	0.29	N/A

**Table 3:** Variant patterns of G6PD and corresponding enzyme activities.

Variant patterns	Class	Male		Female			
		Hemi	Enzyme activity (IU/g·Hb)	Homo	Enzyme activity (IU/g·Hb)	Hete	Enzyme activity (IU/g·Hb)
Valladolid	II	2	1.98	0	—	0	—
NanKang	II	1	1.7	0	—	0	—
Mediterranean	II	1	0.65	0	—	0	—
Coimbra Shunde	II	0	—	0	—	1	2.67
Viangchan	II	73	1.31	4	1.83	6	3.24
Union	II	41	0.66	1	0.12	9	2.8
Canton	II	55	1.26	0	—	5	2.98
Kaiping	II	72	1.32	1	1.28	3	2.13
Gaohe	III	22	1.34	0	—	2	4.15
Orissa	III	2	1.04	1	3.07	0	—
Quing Yan	III	10	2.05	0	—	2	5.19
Chinese-5	III	11	1.38	1	4.14	2	4.19
Taiwan 2	III	1	5.06	0	—	0	—

## Data Availability

The data used to support the findings of this study are included within the article and available from the corresponding author upon request.
